# Geniposide attenuates astrocyte pyroptosis in depression via long non‐coding RNA Six3os1‐mediated regulation of the miR‐511‐3p/COL9A3 axis and MAPK/NLRP3 signaling

**DOI:** 10.1002/ccs3.70043

**Published:** 2025-12-02

**Authors:** Tianyu Zou, Cheng Mei, Xiaoyu Liang, Xiaolong Shang, Guoxiang Duan

**Affiliations:** ^1^ Department of Encephalopathy Shenzhen Luohu District Hospital of Traditional Chinese Medicine Shenzhen China; ^2^ Department of Encephalopathy Heilongjiang Academy of Chinese Medical Sciences Harbin China; ^3^ Department of Acupuncture Heilongjiang Academy of Chinese Medical Sciences Harbin China

**Keywords:** astrocyte pyroptosis, geniposide, long non‐coding RNA Six3os1, MAPK/NLRP3 signaling, miR‐511‐3p

## Abstract

Depression involves multifaceted molecular pathways, with astrocyte pyroptosis emerging as a critical contributor to neuroinflammation. This study reveals that geniposide, a natural compound, alleviates depressive‐like behaviors in chronic unpredictable mild stress mice by targeting a long non‐coding RNA (lncRNA)‐mediated signaling axis. Bioinformatics analysis identified Six3os1 as a key lncRNA sponging miR‐511‐3p, thereby upregulating COL9A3 and suppressing the MAPK/NLRP3 pathway. Behavioral tests (sucrose preference, tail suspension, and Morris water maze) demonstrated that geniposide (100 mg/kg) reversed CUMS‐induced depressive behaviors. Histological and molecular analyses confirmed geniposide's ability to restore hippocampal integrity, reduce astrocyte pyroptosis, and downregulate pyroptosis markers (ASC, cleaved Casp‐1, GSDMD‐N, and IL‐1β). Dual‐luciferase and RNA pull‐down assays validated the Six3os1/miR‐511‐3p/COL9A3 interaction, whereas Western blotting showed geniposide inhibited MAPK phosphorylation (p‐p38, p‐ERK1/2) and NLRP3 activation. Overexpression of Six3os1 or silencing of miR‐511‐3p mimicked geniposide's effects, whereas COL9A3 knockdown exacerbated pyroptosis. These findings establish a novel ceRNA mechanism wherein geniposide modulates astrocyte survival via Six3os1‐dependent regulation of miR‐511‐3p and MAPK/NLRP3 signaling, offering therapeutic insights for depression.

## Graphical abstract

Geniposide ameliorates depressive‐like behaviors in CUMS mice through the lncRNA Six3os1/miR‐511‐3p/COL9A3 axis and MAPK/NLRP3 signaling pathway.

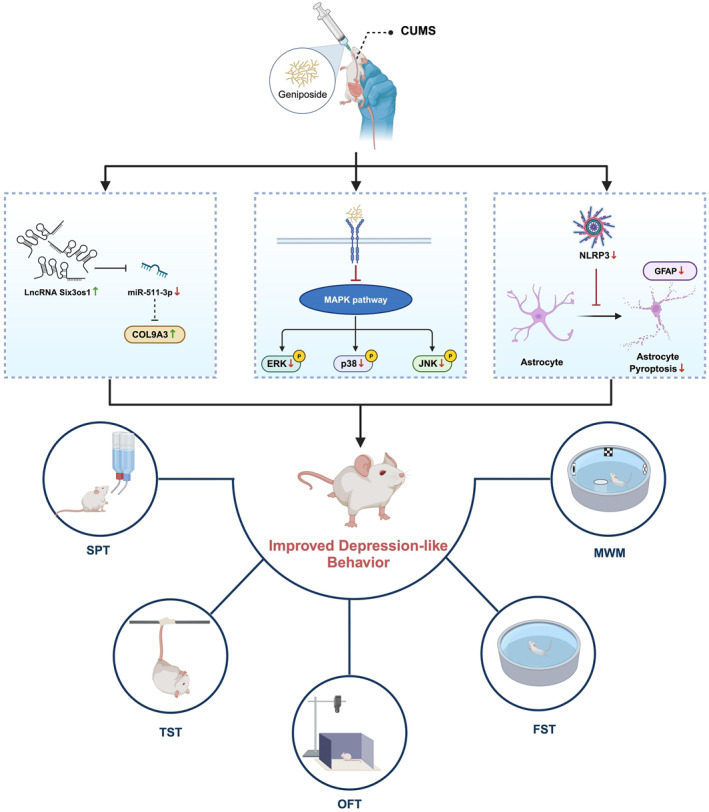

## 1 INTRODUCTION

Depression is a widespread psychiatric condition marked by persistent low mood and reduced interest in activities, often accompanied by fatigue. It affects over 264 million individuals globally and continues to increase in prevalence, ranking among the top contributors to global disease burden.^1^ The etiology of depression is multifactorial, encompassing genetic, biochemical, psychosocial, and environmental influences.^2^ Current neurobiological theories on depression primarily focus on neurotransmitter imbalances, neuroinflammation, and deficiencies in neurotrophic factors.^3^, ^4^, ^5^ However, despite the availability of various antidepressants in clinical use, these medications often take a long time to become effective and are associated with numerous side effects. Therefore, identifying new pathological mechanisms and exploring novel therapeutic targets are crucial for enhancing treatment efficacy and reducing adverse reactions.

In recent years, increasing evidence has highlighted the involvement of neuroglial cells—especially astrocytes—in the pathophysiology of depression. As the predominant glial subtype in the central nervous system, astrocytes contribute significantly to neuronal homeostasis by regulating environmental balance, supporting neurotransmitter turnover, and promoting neurotrophic functions.^6^, ^7^, ^8^ Studies have shown that astrocyte dysfunction in patients with depression may be linked to the metabolism and recycling mechanisms of neurotransmitters such as glutamate and gamma‐aminobutyric acid (GABA).^7^, ^9^ Moreover, the activation of astrocyte pyroptosis alters interneuronal communication and may also exacerbate neuroinflammation by releasing inflammatory cytokines.^10^, ^11^ These cytokines, in turn, can further affect the neural circuits involved in emotion regulation, creating a vicious cycle.^12^ Thus, exploring strategies to control or inhibit astrocyte pyroptosis may hold potential value for the treatment of depression.

In the molecular regulation mechanisms of depression, non‐coding RNAs, particularly long non‐coding RNAs (lncRNAs) and microRNAs (miRNAs), play indispensable roles. LncRNAs modulate RNA stability and activity, thereby influencing cellular behaviors such as proliferation, differentiation, and apoptosis under pathological states.^13^ miRNAs control gene expression post‐transcriptionally by promoting mRNA degradation or inhibiting translation.^14^ In depression models, altered miRNA profiles impact neural cell viability and function by regulating specific downstream genes.^15^ For instance, miR‐511‐3p has been shown to target several genes associated with cell survival and apoptosis.^16^ Although lncRNA Six3os1 has demonstrated potential in regulating crucial biological processes in numerous neurological disorders,^17^ its specific role and mechanisms in depression remain to be fully elucidated.

Geniposide, the principal bioactive compound extracted from Gardenia jasminoides, has been reported to exert anti‐apoptotic and anti‐inflammatory effects.^18^, ^19^ As a novel agonist of the glucagon‐like peptide‐1 receptor (GLP‐1R), geniposide activates GLP‐1R, thereby triggering the PI3K/AKT signaling cascade to suppress hypoxia/reoxygenation‐induced apoptosis.^20^ In streptozotocin‐induced Alzheimer’s disease (AD) models, geniposide treatment significantly attenuates tau phosphorylation, neuronal apoptosis, and memory deficits.^21^ Moreover, in Parkinson’s disease (PD) mouse models, geniposide has been shown to enhance growth factor signaling and inhibit neuronal apoptosis, thereby strengthening anti‐apoptotic activity and reducing blood–brain barrier (BBB) permeability, ultimately protecting neurons from ischemic neurovascular injury.^22^ In addition, geniposide suppresses the release of inflammatory cytokines IL‐6 and TNF‐α from LPS‐stimulated astrocytes.^23^ Further evidence suggests that geniposide modulates both the MAPK signaling pathway and the NLRP3 inflammasome axis. In cardiomyocytes, geniposide significantly inhibits NLRP3 activation and pyroptosis via the AMPK pathway,^24^ supporting its regulatory role in inflammation and cell death processes. The MAPK and NLRP3 inflammasome signaling axes are crucial pathways that regulate cellular responses and inflammatory reactions. In neurological disorders, particularly depression, abnormal activation of these pathways is associated with enhanced cell death and inflammatory responses. The MAPK pathway regulates essential cellular processes such as proliferation, differentiation, and apoptosis.^25^ NLRP3 inflammasome activation contributes to neuroinflammation by inducing inflammatory cytokines, including IL‐1β, which play critical roles in this process. Studies suggest that modulating the MAPK/NLRP3 signaling axis could be beneficial in inhibiting astrocyte pyroptosis and alleviating symptoms of depression.^26^, ^27^, ^28^

In summary, this study aimed to delve into the regulatory effects of geniposide through lncRNA Six3os1 on miR‐511‐3p and COL9A3 expression, and its potential mechanism of inhibiting astrocyte pyroptosis via the MAPK/NLRP3 signaling axis. We established a chronic unpredictable mild stress (CUMS) model of depression in mice to explore the antidepressant effects of geniposide and its molecular mechanisms. The study found that geniposide significantly improved the behavioral performance of the depressive model mice and reduced astrocyte pyroptosis. The mechanism likely involves the upregulation of lncRNA Six3os1, which diminishes miR‐511‐3p and elevates COL9A3 expression, ultimately modulating the MAPK/NLRP3 signaling pathway. These findings not only reveal a potential new mechanism of geniposide in the treatment of depression but also provide new targets for drug development for depression, holding significant scientific and clinical implications. Furthermore, this research supports the theoretical and experimental foundation for using non‐coding RNAs and their related signaling pathways as potential strategies for treating depression.

## 2 MATERIALS AND METHODS

### 2.1 Establishment of a CUMS mouse model with depression‐like phenotypes

Thirty adult male ICR mice (8 weeks old, 19–23 g) were obtained from the Jiangsu Laboratory Animal Center (Nanjing, China). Animals were housed in standard cages under controlled conditions (18–22°C, 12‐h light/dark cycle) with free access to food and water. All procedures were approved by the Institutional Animal Ethics Committee (No. 2023‐LHQZYYYXLL‐KY‐139) and complied with international animal welfare guidelines. Measures were taken to minimize stress and discomfort, and mice were euthanized humanely at the end of the experiment.

Following one week of acclimatization, mice were subjected to CUMS, which included sequential exposure to various stressors: 24 h of food and water deprivation, 5 h of restraint, 8 h of continuous lighting, 20 min of horizontal shaking, 24 h in a tilted cage (45°), and 24 h in a soiled cage containing 500 mL water and 250 g of sawdust. After six weeks of CUMS, behavioral assessments confirmed successful depression‐like phenotypes in all 24 mice.^29^ Control mice (*N* = 6) did not undergo stress procedures. Successfully modeled animals received intraperitoneal injections of 3% pentobarbital sodium (P3761, Sigma‐Aldrich) for deep anesthesia, followed by euthanasia via cervical dislocation. Brains were collected for hippocampal tissue isolation and either used immediately for experiments or snap‐frozen in liquid nitrogen and stored at −80°C.^30^

### 2.2 Prediction of downstream signaling based on miRNA‐Related databases

Utilizing the Human microRNA Disease Database version 4.0 (HMDD) (http://www.cuilab.cn/hmdd), we retrieved and downloaded miRNA precursors listed under the entries “Depressive Disorder” and “Depressive Disorder, Major.” The R language “VennDiagram” package was adopted to identify intersections among these miRNA precursors, yielding a set of intersecting miRNA precursors. Subsequently, potential miRNAs targeting Six3os1 were predicted utilizing the Encyclopedia of RNA Interactomes (ENCORI) (https://rnasysu.com/encori/index.php), and a search was conducted on the intersecting miRNA precursors to identify mature miRNAs associated with depression that could potentially target Six3os1.

Further, miRNA‐511‐3p downstream target genes were collaboratively filtered utilizing ENCORI, miRDB (https://mirdb.org/), and TargetScan (https://www.targetscan.org/mmu_71/) databases. In this process, ENCORI employed a TDMDScore ≥0.95 criterion for filtering predictions, miRDB used a target score ≥85, and TargetScan applied a total context++ score ≤ −0.05. Concurrently, genes related to “Depressive Disorder” were queried from GeneCards (https://www.genecards.org/), selecting the top 1200 genes by relevance score. These genes were then cross‐referenced with the predictions from the three miRNA databases to identify depression‐related downstream target genes of miR‐511‐3p.

### 2.3 Gene functional enrichment analysis and protein‐protein interaction (PPI) network construction

Gene ontology (GO) and Kyoto encyclopedia of genes and genomes (KEGG) enrichment analyses were implemented on genes utilizing the “clusterProfiler” package in R software. Using the GeneMANIA website, a PPI network for the genes and their related genes was constructed.

### 2.4 Mouse treatment groups

Thirty adult male ICR mice (18–22 g) were used and maintained under standard conditions. Following one week of acclimatization, the mice were randomly assigned to five groups (*n* = 6/group): Control, CUMS, CUMS + GP25 (25 mg/kg geniposide), CUMS + GP50 (50 mg/kg), and CUMS + GP100 (100 mg/kg). All groups except the Control group received CUMS treatment for 6 weeks. Following confirmation of modeling success in 24 mice via behavioral testing, daily oral gavage was administered. Control and CUMS groups received equivalent volumes of saline. No signs of toxicity or adverse effects were observed at the tested doses (25, 50, 100 mg/kg). Body weight and sucrose preference were monitored throughout the experiment and remained within normal ranges.

### 2.5 Behavioral testing

To assess depression‐like behaviors, mice were subjected to multiple tests, including the sucrose preference test (SPT), tail suspension test (TST), forced swim test (FST), open field test (OFT), and Morris water maze (MWM).

For the SPT, mice underwent 24 h of food and water deprivation, followed by access to two bottles containing 1% sucrose solution and plain water for 24 h. Sucrose preference was calculated as: (sucrose intake/total fluid intake) × 100%.

In the TST, mice were suspended by the tail from a 15 cm diameter, 30 cm high acrylic rod for 6 min. Immobility during the final 4 min was recorded utilizing a video tracking system (Smart 3.0, Panlab, USA), with increased immobility reflecting depressive‐like behavior.

For the FST, mice were placed in a 20 cm diameter, 50 cm high cylinder filled with water (23–25°C) at a depth preventing tail contact with the bottom. On day one, a 15‐min pre‐swim was performed, followed by a 5‐min test the next day to assess immobility time.

The OFT was conducted in a 40 × 40 × 30 cm plastic chamber. Mice were placed in the center (20 × 20 cm) for 10 min under quiet conditions. Time spent in the central area was automatically recorded utilizing the same tracking system.

In the MWM test, mice were trained in a circular pool (120 cm diameter) maintained at 22 ± 2°C, with a hidden platform submerged 1–2 cm below the water surface. Over five consecutive days, mice were trained to locate the platform to establish spatial memory. Swimming trajectories and escape latency were recorded via an overhead video tracking system. Trials exceeding 120 s were considered unsuccessful. On day six, the platform was removed, and the time spent in the target quadrant was used to evaluate memory retention.

### 2.6 Histological analysis

Following anesthesia, hippocampal tissues were harvested. Hematoxylin and eosin (H&E) staining was used to examine hippocampal morphology. GFAP immunofluorescence staining was performed to quantify astrocytes, and triple labeling with PI, GFAP, and Casp‐1 (p10) was applied to assess astrocyte pyroptosis.

### 2.7 Astrocyte culture

To obtain astrocytes, hippocampi were isolated from neonatal mice under sterile conditions after anesthesia with isoflurane. The bilateral hippocampi were dissected and digested with 0.25% trypsin at 37°C for 10 min. Tissue fragments were then suspended in DMEM enriched with 10% FBS (26010066, Gibco, Thermo Fisher Scientific, USA), 1% penicillin/streptomycin (15140‐122, Thermo Fisher Scientific, USA), and 2 mM L‐glutamine and triturated to form a single‐cell suspension. Astrocytes were filtered using a cell strainer (REF352350, FALCON, USA) and seeded at a density of 4 × 10^6^ cells/well in poly‐D‐lysine (PDL, 100 μg/mL, Sigma‐Aldrich, USA)‐coated six‐well plates. Cells were cultured at 37°C in a 5% CO_2_ incubator, with half of the medium replaced every 2–3 days with glutamate‐free medium. After 3 days, purity was confirmed by GFAP immunofluorescence staining (1:1000, Millipore, Boston, MA, USA), showing >95% astrocyte purity.^31^

### 2.8 Drug treatment

Geniposide (Sigma‐Aldrich, USA) was diluted to final concentrations of 0, 10, 50, and 100 μM. Candidalysin (HY‐P10408, MCE) was diluted to 50 μM based on previous literature.^32^ Candidalysin is a cytolytic peptide toxin secreted by Candida albicans, known to activate the EGFR‐MAPK signaling pathway, induce MMP expression and calcium influx, and modulate c‐Fos and MKP1 via p38 MAPK and ERK1/2 pathways, triggering epithelial immune responses. Candidalysin also activates the NLRP3 inflammasome to initiate inflammatory responses. Astrocytes were treated with geniposide and/or Candidalysin for 24 h. After treatment, cell morphology, viability, proliferation, and pyroptosis were observed utilizing an inverted microscope (Olympus, Japan).

### 2.9 Lentiviral construction

Lentiviral interference (pSIH1‐H1‐copGFP, shRNA) and overexpression (pCDH‐CMV‐MCS‐EF1α‐copGFP) vectors were obtained from System Biosciences (USA) for gene silencing and overexpression. Lentiviruses were produced by transfecting HEK‐293T cells (iCell‐h237, Saibai Kang Biotechnology, Shanghai, China) utilizing a lentiviral packaging kit (A35684CN, Invitrogen, USA). After 48 h, viral supernatants were harvested with a final titer of 1 × 10^8^ TU/mL. shRNA, miRNA inhibitors, and negative controls were all supplied by RiboBio (China).

Astrocytes from the CUMS model were treated as follows: Six3os1 was silenced, miR‐511‐3p was overexpressed, or Six3os1 was silenced while COL9A3 was overexpressed. These astrocytes were incubated with 100 μM geniposide (Sigma‐Aldrich, USA) for 24 h. After incubation, expression of Six3os1, miR‐511‐3p, and COL9A3 was analyzed via qRT‐PCR. Protein expression levels of COL9A3, p‐p38, p‐ERK1/2, NLRP3, IL‐18, ASC, cleaved Casp‐1, GSDMD‐N, and IL‐1β were evaluated by Western blot.

Astrocytes subjected to gene knockdown or overexpression for 48 h were treated with 100 μM geniposide for 24 h. Post‐treatment, an inverted microscope (Olympus, Japan) was used to observe cell morphology, and changes in cell survival, proliferation, and pyroptosis were recorded.

Lentiviral injection: Mice were anesthetized with 3% pentobarbital sodium and secured in a stereotaxic frame. Using a Micro4 microinjection system, 1 μL of lentivirus was bilaterally delivered into the hippocampus (coordinates: 2.2 mm posterior, 2.2 mm lateral, and 1.8 mm dorsal to the bregma) at 300 nL/min. Following the injection, incisions were sutured, and animals were returned to their cages after anesthesia recovery. Behavioral testing commenced 10 days later.

### 2.10 qRT‐PCR gene expression analysis

Total RNA was extracted utilizing Trizol reagent (Invitrogen, USA) following the manufacturer's protocol. cDNA was synthesized with a commercial kit (Thermo Fisher Scientific, USA). Expression levels of Six3os1, miR‐511‐3p, and COL9A3 were quantified utilizing SYBR Green‐based qRT‐PCR (Applied Biosystems, USA).

RNA Extraction: Cells were lysed with 1 mL Trizol and vortexed thoroughly. After standing at room temperature for 5 min, 200 μL chloroform was added and mixed vigorously for 15 s, followed by incubation at room temperature for 3 min. Samples were centrifuged at 12,000 rpm for 15 min, and the upper phase was transferred to a fresh tube. Subsequently, 500 μL isopropanol was added, and samples were incubated at −20°C for 10 min, then centrifuged again at 12,000 rpm for 10 min. The resulting RNA pellet was washed with 75% ethanol, air‐dried, and resuspended in 30 μL RNase‐free water. Reverse transcription was conducted according to the cDNA kit instructions, with a reaction at 37°C for 60 min qRT‐PCR was performed utilizing SYBR Green PCR master mix (Thermo Fisher Scientific, USA). Primers were designed based on published sequences. The relative transcription levels of target genes were calculated utilizing the 2^‐△△CT^ method. Each experiment was performed in triplicate. Primer sequences are detailed in Supporting Information S1: Table S1.

### 2.11 Western blot analysis of protein expression

Cells were lysed in RIPA buffer supplemented with protease inhibitors to extract total protein. Protein content was quantified via the BCA assay. Equal amounts (30 μg) were resolved on 12% SDS‐PAGE gels and electrophoretically transferred to PVDF membranes. Membranes were incubated in 5% skim milk at ambient temperature to block nonspecific binding, followed by overnight exposure to primary antibodies targeting proteins of interest (e.g., Six3os1, Abcam, UK; miR‐511‐3p, Abcam, UK; COL9A3, Cell Signaling Technology, USA; ERK1/2‐ab184699; p‐p38, p‐ERK1/2‐ab50011, NLRP3, IL‐18, ASC, cleaved Casp‐1, GSDMD‐N, and IL‐1β, Abcam, UK). After washes, HRP‐conjugated secondary antibodies were applied for signal detection, which was performed utilizing an enhanced chemiluminescence system.

Detailed procedure: lysis: Cell pellets were incubated in 200 μL RIPA buffer on ice for 30 min, centrifuged at 12,000 rpm (10 min), and supernatants collected. Electrophoresis: Protein samples were run at 100 V for 90 min on precast 12% gels. Transfer: Blotting onto PVDF membranes was performed at 300 mA for 90 min. Blocking & probing: After blocking, membranes were incubated sequentially with primary (overnight, 4°C) and secondary antibodies (1 h, room temperature). Visualization: Signal development was carried out with ECL reagents, and band intensities were analyzed.

### 2.12 Dual‐luciferase reporter gene assay

To assess interactions among Six3os1, miR‐511‐3p, and COL9A3, a dual‐luciferase system was employed. The 3′UTR sequences of Six3os1 and COL9A3 were cloned into the pmir‐REPORT vector (Promega, USA) to generate both wild‐type and mutant constructs.

Cell transfection: HEK293T cells were co‐transfected with reporter plasmids and either miR‐511‐3p mimic or mimic NC utilizing Lipofectamine 3000 (Thermo Fisher Scientific, USA).

Luciferase measurement: At 48 h post‐transfection, luciferase activity was quantified utilizing the Dual‐Luciferase Reporter Assay System (Promega, USA). Briefly, cells were lysed in 100 μL of 1× passive lysis buffer and shaken at 4°C for 15 min. For firefly luciferase detection, 20 μL of lysate was combined with 100 μL Luciferase Assay Reagent II (LAR II). Then, 100 μL Stop & Glo Reagent was added to the same sample to determine Renilla luciferase activity.

### 2.13 RNA immunoprecipitation (RIP)

A RIP assay kit (Millipore, USA) assessed the interactions between Six3os1, COL9A3, and the AGO2 protein. The detailed steps are as follows:

Cell preparation and lysis: Upon reaching 80%–90% cell confluency, the medium was discarded and monolayers rinsed with 1 mL cold PBS. Cells were then lysed in 1 mL RIP lysis buffer and kept on ice for 5 min. Lysates were centrifuged at 14,000 rpm for 10 min at 4°C, and the supernatants were collected for subsequent immunoprecipitation.

Immunoprecipitation: Magnetic beads (50 μL) were washed and resuspended in 100 μL RIP wash buffer. For each reaction, 5 μg AGO2 antibody (Millipore, USA) was added, and the mixture was incubated overnight at 4°C. The antibody‐coated beads were then mixed with 900 μL RIP wash buffer and 100 μL cell lysate, followed by another overnight incubation at 4°C. Beads were isolated utilizing a magnetic rack and washed three times.

RNA extraction: To extract RNA, 200 μL proteinase K buffer was added to the bead complexes and incubated at 37°C for 30 min. Purified RNA was then subjected to qRT‐PCR to determine Six3os1 and COL9A3 expression levels.

### 2.14 RNA pull‐down assay

To investigate the interaction between miR‐511‐3p, Six3os1, and COL9A3, RNA pull‐down assays were conducted. Biotin‐labeled RNAs (Bio‐NC, Bio‐Six3os1‐WT, Bio‐Six3os1‐Mut, Bio‐COL9A3‐WT, and Bio‐COL9A3‐Mut; RiboBio, China) were transfected into astrocytes utilizing Lipofectamine 3000 (Thermo Fisher Scientific, USA) at a final concentration of 50 nM and incubated for 48 h. Following PBS washing, cells were harvested and lysed utilizing a commercial buffer (Ambion, USA) for 10 min. Lysates were incubated with M‐280 streptavidin‐coated magnetic beads (Invitrogen, USA), pre‐blocked with RNase‐free water and yeast tRNA, for 3 h at room temperature. Beads were sequentially washed with low‐salt buffer (twice) and high‐salt buffer (once). Bound RNAs were extracted utilizing Trizol and analyzed by qRT‐PCR to assess miR‐511‐3p enrichment.

### 2.15 H&E staining

Hippocampal tissues and hepatic tissues were collected from mice after anesthesia and euthanasia. The tissues were fixed in a 10% neutral formalin solution, embedded in paraffin, and sectioned at a thickness of 4 μm. H&E staining involved hematoxylin staining for 5 min, followed by rinsing with running water. Sections were differentiated utilizing 1% hydrochloric acid–alcohol, reblued in hematoxylin, and counterstained with eosin for 2 min. Structural changes in the hippocampal tissue and hepatic tissues were observed under a microscope.

### 2.16 Immunofluorescence staining

Frozen brain sections (10 μm) were treated with 0.3% Triton X‐100 for permeabilization and blocked with 5% BSA for 1 h. Primary antibodies (e.g., GFAP, 1:500, Sigma‐Aldrich, USA) were applied for overnight incubation (4°C) for sections. The next day, fluorescent secondary antibodies (1:1000, Invitrogen, USA) were introduced, followed by 1‐h incubation at room temperature in the dark. Nuclei were stained with DAPI, and fluorescence images were acquired utilizing a confocal microscope (Leica, Germany).

### 2.17 Data analysis

All experiments were performed in triplicate, and results are summarized as mean ± standard deviation. Statistical analysis was completed utilizing GraphPad Prism 8 (GraphPad Software, USA). Group comparisons were made via one‐way ANOVA, with *p* < 0.05 established as statistically significant.

## 3 RESULTS

### 3.1 Geniposide May regulate the miR‐511‐3p/COL9A3 signaling axis via lncRNA Six3os1

Our previous research demonstrated that geniposide alleviates depressive‐like behaviors in CUMS mice by upregulating lncRNA Six3os1.^29^, ^33^ However, the precise underlying mechanism remains unclear. The interaction between lncRNAs and miRNAs is a critical regulator of gene expression and plays a significant role in depression.^34^, ^35^

**FIGURE 1:**
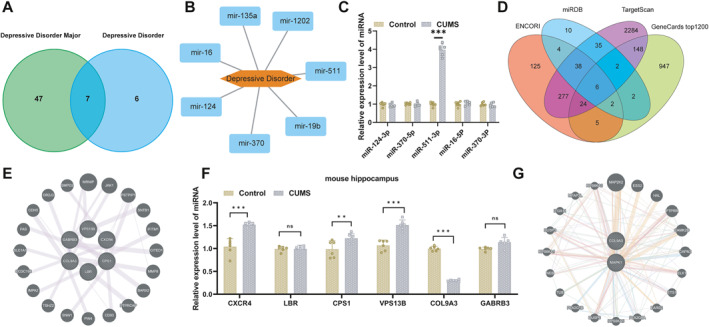
Downstream signaling pathway screening for the Geniposide/Six3os1 axis in depression mice. (A, B) Overlap of miRNA precursors from the HMDD database entries “Depressive Disorder” and “Depressive Disorder, Major” with their visualization; (C) RT‐qPCR analysis of five miRNAs in the hippocampal tissue of Control and CUMS mice; (D) intersection of miR‐511‐3p downstream target genes predicted by ENCORI, miRDB, and TargetScan databases with top 1200 “Depressive Disorder” genes from the GeneCards database; (E) interaction network of six intersecting genes and related genes obtained from the GeneMANIA database; (F) RT‐qPCR analysis of the expression of six related genes in the hippocampal tissue of Control and CUMS mice; and (G) interaction network of COL9A3 and its related genes obtained from the GeneMANIA database. Animal experiment groups: *n* = 6 per group. CUMS, chronic unpredictable mild stress. Statistical significance: ^ns^ *p* > 0.05, *p < 0.05, **p < 0.01, and ***p < 0.001.

To explore the potential involvement of Six3os1‐targeting miRNAs and their functional networks, we conducted analyses based on public databases such as HMDD (Supporting Information S1: Figure S1). To identify miRNAs potentially mediating the geniposide/Six3os1 axis in alleviating depressive‐like behaviors in CUMS mice, we retrieved depression‐related miRNAs from the HMDD database. The “Depressive Disorder” category included 13 miRNA precursors, whereas the “Depressive Disorder, Major” category contained 54 miRNA precursors. The intersection of these two datasets revealed seven overlapping miRNA precursors: mir‐19b, mir‐370, mir‐124, mir‐511, mir‐135a, mir‐16, and mir‐1202 (Figure 1A,B).

Using the ENCORI database, we predicted miRNAs targeting Six3os1 and analyzed the corresponding mature forms of seven depression‐related miRNA precursors. The results indicated that miR‐370‐5p (TDMDScore = 1.4085), miR‐370‐3p (TDMDScore = 0.9945), miR‐124‐3p (TDMDScore = 1.5268), miR‐511‐3p (TDMDScore = 1.2383), and miR‐16‐5p (TDMDScore = 0.7006) are associated with depression and potentially target Six3os1.

Subsequently, we established the CUMS mouse model and analyzed the expression of five miRNA mature forms (miR‐124‐3p, miR‐370‐5p, and miR‐511‐3p, miR‐16‐5P, and miR‐370‐3P) in the hippocampal tissue of CUMS mice. The results showed that miR‐511‐3p expression was notably elevated in CUMS mice compared to Control mice, whereas the expression levels of miR‐124‐3p, miR‐16‐5P, miR‐370‐3P, and miR‐370‐5p did not differ significantly between the two groups (Figure 1C). Based on these findings, we hypothesize that the geniposide/Six3os1 axis alleviates depressive‐like behaviors in CUMS mice by modulating miR‐511‐3p.

To further investigate the downstream genes and signaling pathways of the Six3os1/miR‐511‐3p axis, we predicted the target genes of miR‐511‐3p using three databases described in the Methods section. The ENCORI database identified 481 genes, the miRDB database identified 99 genes, and the TargetScan database identified 2814 genes. Additionally, 1200 genes associated with “Depressive Disorder” were retrieved from the GeneCards database based on their Relevance score, and the intersection of these datasets was analyzed. Six potential depression‐related target genes of miR‐511‐3p were identified: CXCR4, LBR, CPS1, VPS13B, COL9A3, and GABRB3 (Figure 1D).

PPI network construction and functional enrichment analyses revealed that these six genes were primarily enriched in the urea cycle, response to histamine and midgut development GO terms, as well as the nitrogen metabolism, steroid biosynthesis, and arginine biosynthesis pathways (Figure 1E, Supporting Information S1: Figure S2A,B). To prioritize key candidates, the expression of these genes was examined in the hippocampus. RT‐qPCR analysis showed that, relative to controls, CXCR4, CPS1, and VPS13B were notably upregulated in the hippocampus of CUMS mice, whereas COL9A3 expression was diminished. In contrast, LBR and GABRB3 levels remained unchanged between groups (Figure 1F).

Because miRNAs typically suppress the expression of their target genes, we hypothesize that miR‐511‐3p predominantly regulates COL9A3 in CUMS mice. Furthermore, GeneMANIA interaction analysis indicated a strong association between COL9A3 and MAPK‐related signaling pathways, suggesting potential regulatory interactions (Figure 1G).

Collectively, geniposide alleviates depressive‐like behaviors in CUMS mice by enhancing Six3os1 expression, potentially via the miR‐511‐3p/COL9A3/MAPK signaling axis.

### 3.2 Geniposide improves depressive‐like behaviors in CUMS mice

The experimental workflow for the in vivo mouse study is illustrated in Supporting Information S1: Figure S3. Optimal dosages of geniposide were determined through behavioral tests and biochemical indicators, followed by bioinformatics analysis to identify key targets influencing depressive‐like behaviors in mice.

**FIGURE 2:**
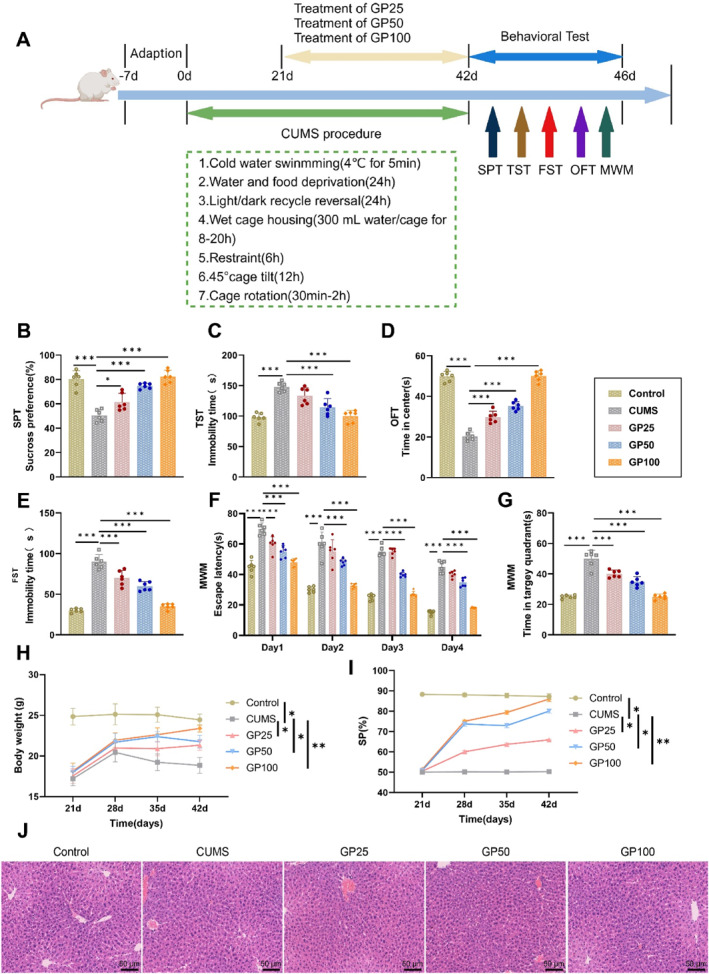
Effects of geniposide on depressive‐like behaviors in CUMS mice. (A) Behavioral test timeline; (B) sucrose preference test evaluating the effect of different doses of geniposide on sucrose preference percentage in CUMS mice; (C) tail suspension test measuring the effect of geniposide treatment on immobility time in CUMS mice; (D) open field test assessing the effect of geniposide treatment on time spent in the central area by CUMS mice; (E) forced swim test measuring the impact of geniposide on immobility time in CUMS mice; (F) Morris water maze test recording the average time spent escaping the platform (escape latency) during training days across groups; (G) average time spent in the target quadrant during the 1‐min probe trial; (H) weekly body weight of mice in each group was recorded from day 21 to day 42; (I) weekly sucrose preference index was measured from day 21 to day 42 in all groups; and (J) H&E staining was performed to evaluate liver morphology across groups. Data are presented as mean ± standard error. CUMS, chronic unpredictable mild stress. Statistical significance: *p < 0.05, **p < 0.01, and ***p < 0.001. *N* = 6.

The timeline for behavioral testing in mice is shown in Figure 2A. In this study, CUMS mice were treated with different doses of geniposide, and a series of behavioral experiments were conducted. In the SPT, geniposide significantly increased the sucrose preference percentage in CUMS mice, with the GP100 group showing the highest sucrose preference, approaching levels observed in the Control group (Figure 2B). Similarly, in the TST, geniposide treatment distinctly reduced immobility time in CUMS mice, with the GP100 group displaying immobility times comparable to the Control (Figure 2C).

The OFT results showed that geniposide treatment significantly increased the time spent in the central area, with the GP100 group demonstrating significantly higher activity in the center than other treatment groups (Figure 2D). In the FST, geniposide treatment notably reduced the immobility time of CUMS mice, with the GP100 group exhibiting the lowest immobility time among all groups (Figure 2E). In the MWM test, geniposide treatment significantly shortened the escape latency, with the GP100 group showing a markedly shorter latency than other treatment groups. Additionally, the time spent in the target quadrant by the GP100 group was comparable to that of the Control group (Figure 2F,G). Body weight analysis showed that geniposide treatment increased the weight and sucrose preference index of CUMS mice. The GP100 group exhibited significantly greater body weight and sucrose preference than other treatment groups, indicating improved appetite and mood (Figure 2H,I). H&E staining of liver tissues showed no pathological changes in any group, suggesting that geniposide, even at high doses, did not induce hepatic toxicity (Figure 2J).

In summary, geniposide demonstrated significant antidepressant and cognition‐enhancing effects, providing strong support for its potential application in the treatment of mood disorders.

### 3.3 Geniposide attenuates CUMS‐Induced damage by promoting COL9A3 expression via the Six3os1/miR‐511‐3p axis

**FIGURE 3:**
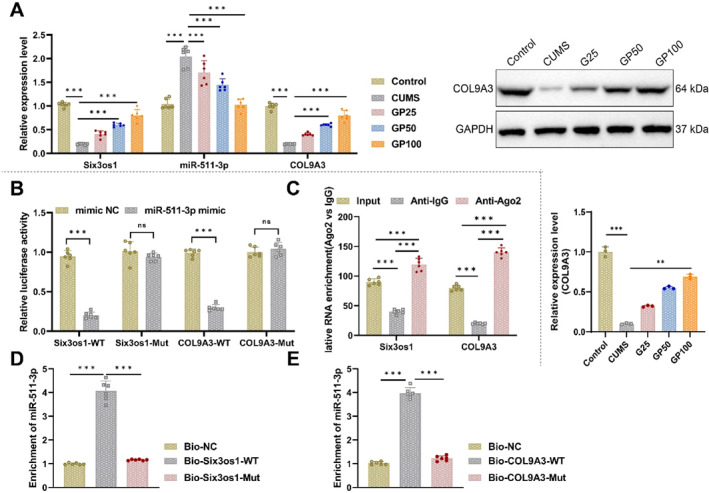
Expression and regulation of the Six3os1/miR‐511‐3p/COL9A3 axis in the hippocampus of CUMS mice. (A) qRT‐PCR analysis of Six3os1, miR‐511‐3p, and COL9A3 expression and Western blot analysis of COL9A3 protein expression in the hippocampus of CUMS mice (*N* = 6); (B) Dual‐luciferase reporter assay evaluating the targeting relationship between miR‐511‐3p, COL9A3, and Six3os1; (C) RIP assay examining the binding of Six3os1 and COL9A3 with AGO2 protein; (D) qRT‐PCR analysis of the enrichment of miR‐511‐3p on biotin‐labeled Six3os1; and (E) qRT‐PCR analysis of the enrichment of miR‐511‐3p on biotin‐labeled COL9A3. For animal experiments, *N* = 6; data were collected from three independent replicates for cellular experiments. Data are presented as mean ± standard error. CUMS, chronic unpredictable mild stress. Statistical significance: *p < 0.05, **p < 0.01, and ***p < 0.001.

qRT‐PCR analysis of hippocampal tissue revealed that geniposide, particularly in the GP100 group, significantly increased Six3os1 and COL9A3 expression while reducing miR‐511‐3p levels compared to the CUMS group. Western blot results further confirmed elevated COL9A3 protein expression in geniposide‐treated mice (Figure 3A).

To investigate the underlying mechanism, in vitro experiments were conducted as outlined in Supporting Information S1: Figure S4. Wild‐type and mutant COL9A3 plasmids were generated and co‐transfected into HEK293T cells alongside miR‐511‐3p mimics or negative controls. Luciferase assays were used to assess target gene activity, and RIP was performed to examine RNA‐protein interactions.

Additionally, astrocytes were isolated from the animal model, and immunofluorescence, knockdown, and overexpression experiments were conducted to determine whether geniposide exerts its antidepressant effects by regulating astrocyte pyroptosis.

First, HEK293T cells validated the regulatory relationship between geniposide and the Six3os1/miR‐511‐3p/COL9A3 axis through dual‐luciferase reporter assays and RIP experiments. As shown in Figure 3B–E, dual‐luciferase assays demonstrated that co‐transfection of miR‐511‐3p mimic with Six3os1‐WT plasmids distinctly reduced luciferase activity compared to co‐transfection of mimic NC with Six3os1‐WT plasmids. In contrast, no significant difference in luciferase activity was observed between the miR‐511‐3p mimic and mimic NC groups co‐transfected with Six3os1‐Mut plasmids. Similarly, dual‐luciferase assays for COL9A3 showed the same trend (Figure 3B).

RIP assays were performed to assess the binding of Six3os1 and COL9A3 to the AGO2 protein. The results revealed significant binding of both Six3os1 and COL9A3 to AGO2 (Figure 3C).

Next, we further validated the direct binding of miR‐511‐3p to Six3os1 and COL9A3. HEK293T cells were transfected with biotin‐labeled RNA constructs, including Bio‐NC, Bio‐Six3os1‐WT/Mut, and Bio‐COL9A3‐WT/Mut (50 nM). qRT‐PCR analysis showed that miR‐511‐3p was notably enriched in the WT groups of both Six3os1 and COL9A3, whereas no significant enrichment was observed in the Mut groups (Figure 3D,E).

These findings, combined with earlier results, suggest that geniposide regulates the Six3os1/miR‐511‐3p/COL9A3 axis by competitively binding to miR‐511‐3p, thereby inhibiting its negative regulation of COL9A3 and promoting COL9A3 expression.

### 3.4 Six3os1 reduces astrocyte pyroptosis by sponging miR‐511‐3p to target COL9A3 and regulate the MAPK/NLRP3 pathway

The MAPK signaling pathway is critical in cell survival and inflammatory responses, including pyroptosis, through activation of the NLRP3 inflammasome. As such, the MAPK/NLRP3 axis is a central regulator of neuroinflammation and pyroptosis.^29^, ^36^

Based on previous bioinformatics analyses, we identified that the lncRNA Six3os1 is highly expressed in astrocytes and functions as a molecular sponge for miR‐511‐3p, thereby liberating its downstream target gene, COL9A3, to regulate the MAPK/NLRP3 signaling pathway. These findings offer new insights into how astrocyte pyroptosis is triggered by CUMS.

**FIGURE 4:**
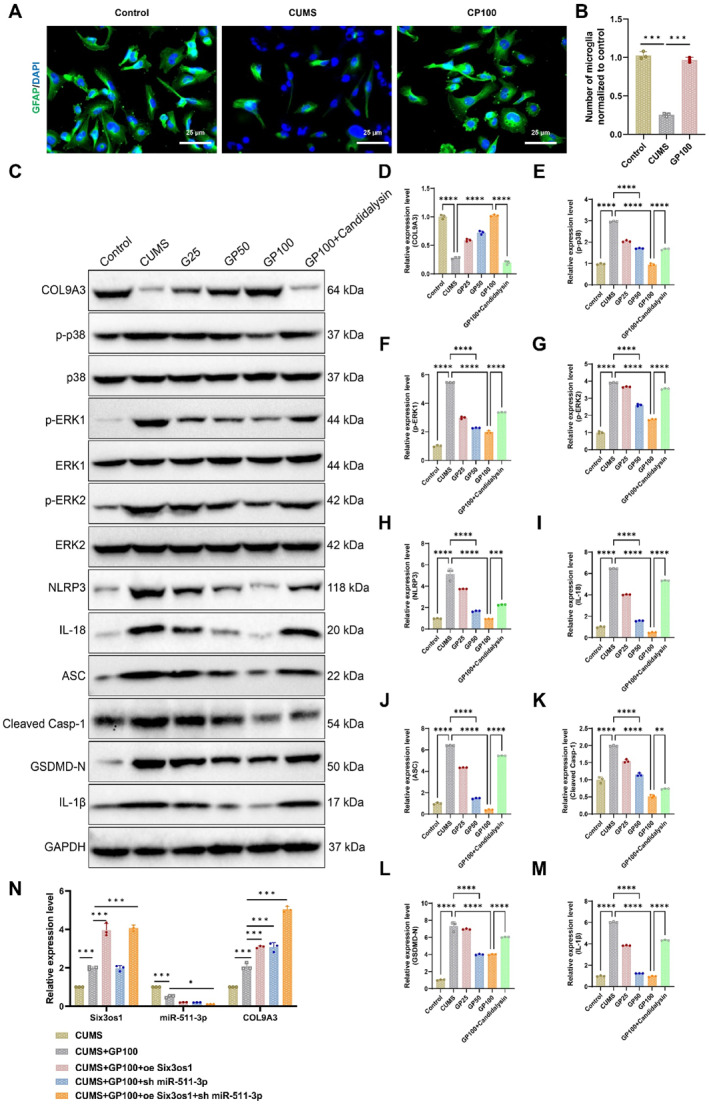
Analysis of geniposide regulation of astrocyte pyroptosis and its mechanisms. (A) Immunofluorescence staining (GFAP, green) assessing astrocyte purity in Control and CUMS groups; (B) statistical analysis of astrocyte purity across groups; (C) Western blot analysis of COL9A3, p‐p38, p‐ERK1/2, NLRP3, IL‐18, ASC, cleaved Casp‐1, GSDMD‐N, and IL‐1β protein expression in astrocytes from CUMS and geniposide‐treated groups and Candidalysin groups; (D–M) relative protein expression levels for the following: (D) COL9A3; (E) p‐p38; (F) p‐ERK1; (G) p‐ERK2; (H) NLRP3; (I) IL‐18; (J) ASC; (K) cleaved Casp‐1; (L) GSDMD‐N; and (M) IL‐1β. (N) qRT‐PCR analysis of Six3os1, miR‐511‐3p, and COL9A3 expression in astrocytes from the CUMS model, evaluating the effects of Six3os1 overexpression or miR‐511‐3p inhibition on COL9A3 expression. Data are presented as mean ± standard error. CUMS, chronic unpredictable mild stress. Statistical significance: *p < 0.05, **p < 0.01, and ***p < 0.001. *N* = 3.

In this study, astrocytes were isolated from the hippocampal tissues of successfully modeled mice (Control, CUMS, and GP100 groups). After culturing under specified conditions for 3 days, the astrocytes were subjected to GFAP (green) immunofluorescence staining. The results confirmed that the purity of astrocytes was approximately 95% (Figure 4A,B).

To determine whether geniposide mitigates depression by modulating pyroptosis, Western blot analysis was performed on astrocytes to assess the protein levels of COL9A3, p‐p38, p‐ERK1/2, NLRP3, IL‐18, ASC, cleaved Casp‐1, GSDMD‐N, and IL‐1β. The results revealed that, compared to the Control group, COL9A3 protein expression was evidently downregulated in the CUMS group, whereas the other proteins were notably upregulated. Geniposide treatment, particularly in the GP100 group, reversed these changes by significantly upregulating COL9A3 and downregulating the expression of the other proteins. Compared to the GP100 group, treatment with GP100 combined with Candidalysin led to significantly increased expression of all proteins except COL9A3, which remained downregulated in the CUMS group (Figure 4C–M). These findings suggest that geniposide alleviates depression by modulating pyroptosis through the MAPK/NLRP3 pathway.

In astrocytes derived from the CUMS model, gene overexpression and silencing experiments were conducted to examine the expression levels of Six3os1, miR‐511‐3p, and COL9A3. qRT‐PCR results showed that overexpression of Six3os1 or inhibition of miR‐511‐3p significantly upregulated COL9A3 expression. Furthermore, simultaneous overexpression of Six3os1 and silencing of COL9A3 further enhanced COL9A3 expression (Supporting Information S1: Figure S5A).

Western blot analysis confirmed that overexpression of Six3os1 or inhibition of miR‐511‐3p increased COL9A3 protein levels while reducing the expression of p‐p38, p‐ERK1/2, NLRP3, IL‐18, ASC, cleaved Casp‐1, GSDMD‐N, and IL‐1β. Moreover, simultaneous overexpression of Six3os1 and silencing of COL9A3 further elevated COL9A3 expression and significantly suppressed the levels of the pyroptosis‐related proteins mentioned above (Figures S5B‐L).

Astrocytes from the CUMS model were treated with 100 μM geniposide for 24 h, followed by overexpression of Six3os1, inhibition of miR‐511‐3p, or simultaneous overexpression of Six3os1 and inhibition of miR‐511‐3p. qRT‐PCR results demonstrated that geniposide treatment significantly upregulated Six3os1 expression, downregulated miR‐511‐3p, and upregulated COL9A3 (Figure 4N).

**FIGURE 5:**
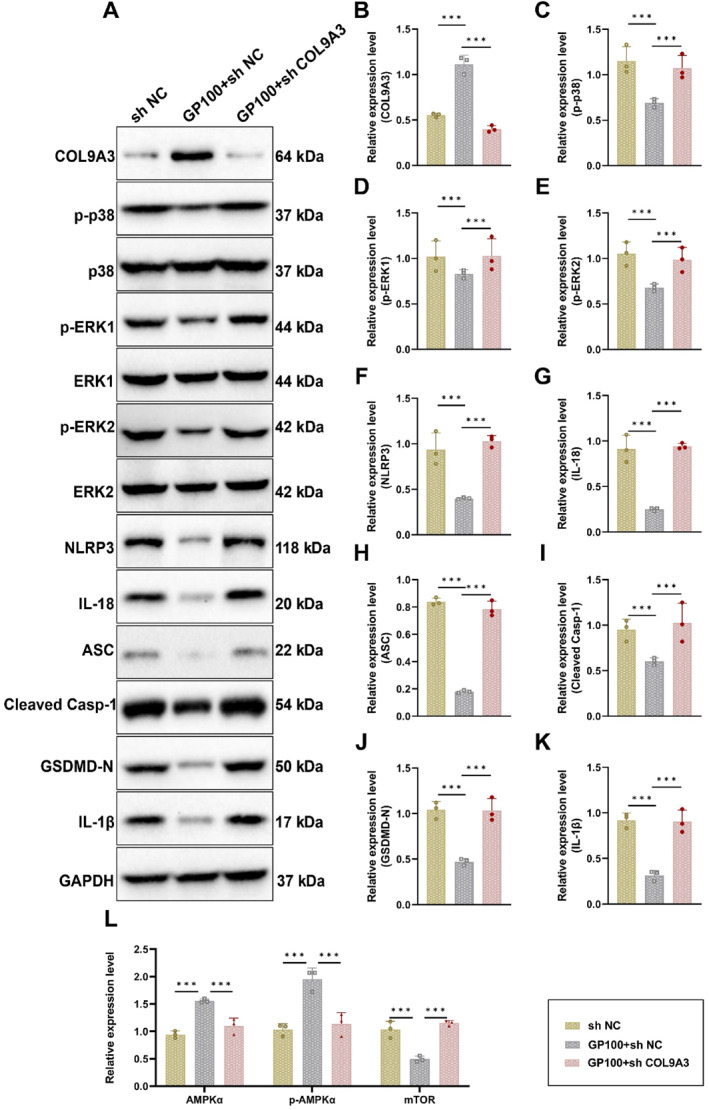
Silencing COL9A3 gene expression activates the MAPK signaling pathway. (A) Western blot analysis of the effects of various treatments on the expression levels of COL9A3, p‐p38, p‐ERK1/2, NLRP3, IL‐18, ASC, cleaved Casp‐1, GSDMD‐N, and IL‐1β in astrocytes from the chronic unpredictable mild stress model; (B–K) relative protein expression levels across groups: (B) COL9A3; (C) p‐p38; (D) p‐ERK1; (E) p‐ERK2; (F) NLRP3; (G) IL‐18; (H) ASC; (I) cleaved Casp‐1; (J) GSDMD‐N; and (K) IL‐1β; (L) qRT‐PCR analysis of AMPK pathway‐related protein expression in astrocytes following geniposide treatment. Data are presented as mean ± standard error. Statistical significance: *p < 0.05, **p < 0.01, and ***p < 0.001. *N* = 3.

The GENEMANIA database identified a regulatory relationship between COL9A3 and MAPK (Figure 1G). Literature evidence further suggests that silencing COL9A3 activates the MAPK signaling pathway. Western blot analysis revealed that silencing COL9A3 after geniposide treatment significantly increased the expression levels of p‐p38, p‐ERK1/2, NLRP3, IL‐18, ASC, cleaved Casp‐1, GSDMD‐N, and IL‐1β (Figure 5A–K).

To investigate whether the Six3os1/miR‐511‐3p/COL9A3 axis regulates the MAPK/NLRP3 signaling pathway through the AMPK pathway, AMPK‐related protein expression levels were analyzed in astrocytes following geniposide treatment. qRT‐PCR results showed that geniposide significantly upregulated AMPKα and p‐AMPKα expression while downregulating mTOR. However, knocking down COL9A3 reversed these effects (Figure 5L). These findings suggest that geniposide exerts its antidepressant effects by activating the AMPK pathway, inhibiting the mTOR pathway and ultimately modulating the MAPK/NLRP3 pathway.

In conclusion, these results confirm that geniposide alleviates depressive‐like behaviors by regulating the Six3os1/miR‐511‐3p/COL9A3 axis to influence the MAPK/NLRP3 signaling pathway.

### 3.5 Geniposide alleviates depressive‐like behaviors in mice by modulating the MAPK/NLRP3 pathway via the Six3os1/miR‐511‐3p/COL9A3 axis

The CUMS‐induced depression mouse model was constructed, and behavioral tests were conducted. The mice were divided into four groups: sh NC + oe NC, GP100+sh NC + oe NC, GP100+sh Six3os1+oe NC, and GP100+sh Six3os1+oe COL9A3. This study investigated the effects of geniposide on depressive‐like behaviors in mice through its regulation of the Six3os1/miR‐511‐3p/COL9A3 axis and the MAPK/NLRP3 pathway.

**FIGURE 6:**
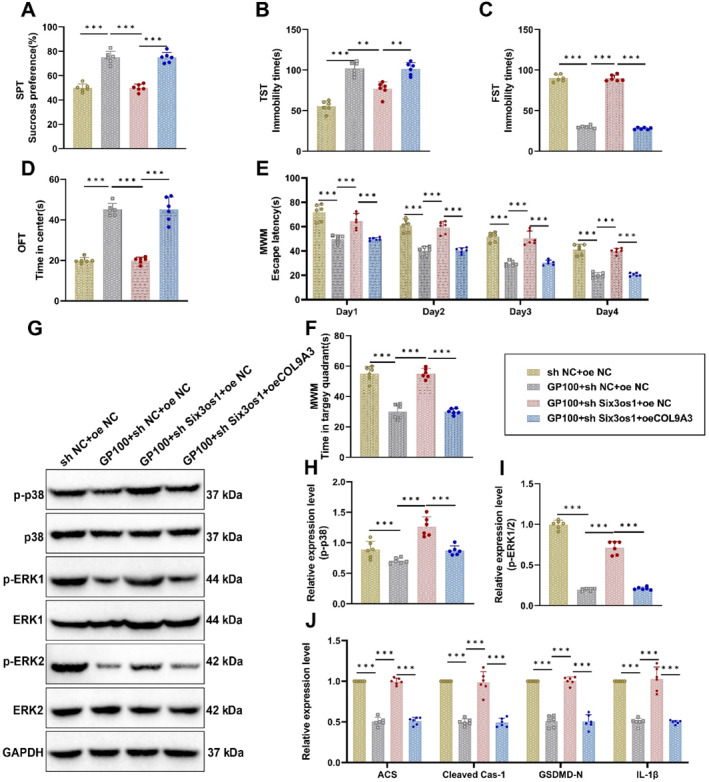
Geniposide modulates MAPK/NLRP3 signaling via the Six3os1/miR‐511‐3p/COL9A3 axis to influence behavioral and histopathological changes in depression model mice. (A) Sucrose preference test evaluating sucrose consumption in CUMS, CUMS + GP100, CUMS + GP100+sh Six3os1, and CUMS + GP100+sh Six3os1+oeCOL9A3 groups; (B) tail suspension test assessing immobility time across groups; (C) forced swim test measuring immobility time across groups; (D) open field test evaluating time spent in the central zone for different groups; (E) Morris water maze test tracking escape latency during training sessions across groups; (F) time spent in the target quadrant during the 1‐min probe trial; (G) Western blot analysis of p‐p38 and p‐ERK1/2 expression levels in the hippocampus of CUMS, CUMS + GP100, CUMS + GP100+sh Six3os1, and CUMS + GP100+sh Six3os1+oeCOL9A3 groups; (H) relative protein expression levels of p‐p38 across groups; (I) relative protein expression levels of p‐ERK1/2 across groups; and (J) qRT‐PCR analysis of mRNA levels for ASC, cleaved Casp‐1, GSDMD‐N, and IL‐1β in the hippocampus of CUMS, CUMS + GP100, CUMS + GP100+sh Six3os1, and CUMS + GP100+sh Six3os1+oeCOL9A3 groups. Data are presented as mean ± standard error. CUMS, chronic unpredictable mild stress. Statistical significance: *p < 0.05, **p < 0.01, and ***p < 0.001. *N* = 6.

Behavioral tests demonstrated that CUMS mice exhibited significant depressive‐like behaviors, including reduced sucrose consumption in the SPT (Figure 6A), increased immobility time in the TST and FST (Figure 6B,C), and decreased locomotor activity in the OFT and MWM (Figure 6D–F). In contrast, the geniposide treatment group (CUMS + GP100) showed marked improvements across all behavioral tests, with these behavioral parameters largely restored to normal levels. However, knockdown of Six3os1 significantly attenuated the therapeutic effects of geniposide, whereas overexpression of COL9A3 partially restored the improvements.

Further analysis of the effects of geniposide on the MAPK signaling pathway showed that the expression levels of p‐p38 and p‐ERK1/2 were significantly upregulated in the hippocampal tissues of CUMS mice, indicating enhanced MAPK pathway activity. Geniposide treatment (CUMS + GP100) markedly reduced the expression of p‐p38 and p‐ERK1/2, demonstrating its ability to inhibit excessive MAPK pathway activation. However, the knockdown of Six3os1 (GP100+sh Six3os1) weakened the inhibitory effect of geniposide on p‐p38 and p‐ERK1/2. Overexpression of COL9A3 (GP100+sh Six3os1+oe COL9A3) significantly restored the suppression of p‐p38 and p‐ERK1/2, indicating that oe COL9A3 could further inhibit MAPK pathway activity (Figure 6G–I).

Additionally, qRT‐PCR was used to measure the mRNA levels of pyroptosis‐related proteins (ASC, cleaved Casp‐1, GSDMD‐N, and IL‐1β) in the hippocampal tissues of mice. Compared to the CUMS group, geniposide treatment diminished the mRNA expression levels of these proteins, indicating its ability to suppress pyroptosis. However, the knockdown of Six3os1 led to a notable increase in the mRNA levels of these proteins, which was partially restored by overexpression of COL9A3 (Figure 6J).

In conclusion, geniposide exerts its potential antidepressant effects in the CUMS mouse model by regulating the Six3os1/miR‐511‐3p/COL9A3 axis. This regulation modulates the MAPK/NLRP3 signaling pathway, suppressing MAPK activity and NLRP3 inflammasome formation, thereby providing insights into its underlying mechanism of action against depression.

## 4 DISCUSSION

Depression is a prevalent mental disorder that severely impacts human health, and its complex pathological mechanisms remain a focus of neuroscience research. Although various antidepressant medications are available, a significant proportion of patients fail to respond adequately to these treatments or experience severe side effects.^37^, ^38^, ^39^ Consequently, identifying novel therapies and mechanisms of action holds immense clinical significance. Emerging evidence has documented the crucial role of astrocytes in depression,^40^ particularly their involvement in pyroptosis, which is closely associated with pathological conditions.^26^, ^41^, ^42^ This study investigates the role of geniposide in upregulating lncRNA Six3os1 to inhibit the MAPK/NLRP3 signaling axis and regulate astrocyte pyroptosis in a mouse model of depression, addressing a gap in the current literature.

Our findings demonstrate a close relationship between the antidepressant effects of geniposide and its regulation of astrocyte pyroptosis. Experimental results revealed that geniposide significantly upregulates the expression of lncRNA Six3os1 while reducing the expression of pyroptosis‐related proteins such as cleaved Casp‐1 and GSDMD‐N. These results confirm the significance of geniposide in cell death pathways and underscore its ability to influence neuropathological states through specific molecular mechanisms. Unlike previous studies that focused on the antioxidant and anti‐inflammatory properties of geniposide, our research further elucidates its role in modulating specific lncRNAs and miRNAs in the nervous system, providing a new direction for exploring the mechanisms of action of antidepressant drugs.

This study highlights the critical role of lncRNA Six3os1 in the pathological mechanisms of depression, demonstrating that it positively regulates COL9A3 by suppressing miR‐511‐3p, thereby modulating the MAPK/NLRP3 signaling axis. This finding aligns with previous research indicating that lncRNAs can regulate miRNA activity through the competing endogenous RNA (ceRNA) mechanism to influence neural function.^43^ However, this is the first study to uncover the involvement of Six3os1 in regulating a specific signaling pathway associated with astrocyte pyroptosis, providing a novel perspective on the complex molecular networks underlying depression. Additionally, this study is the first to report the potential protective role of COL9A3 in depression, identifying it as a promising therapeutic target.

Our results demonstrate that geniposide further modulates the MAPK/NLRP3 signaling axis by influencing the expression of miR‐511‐3p and COL9A3, which play a significant role in controlling astrocyte pyroptosis and the overall inflammatory response. Previous studies have established that activation of the MAPK/NLRP3 signaling axis exacerbates cell damage and pathological conditions in various inflammation‐related diseases. This study demonstrates the potential to suppress this signaling axis by targeting upstream molecules, offering a new strategy to treat depression by modulating micro‐molecular regulatory networks.

Mice treated with geniposide exhibited significant behavioral improvements in tests such as the SPT and TST. Additionally, histological analysis utilizing H&E staining and GFAP immunofluorescence revealed that geniposide improved hippocampal structure and reduced the number of astrocytes. These findings not only confirm geniposide's antidepressant effects at the behavioral level but also demonstrate its regulatory impact on neuropathological states at the cellular level. Compared to other studies, these results highlight the possibility that geniposide exerts its effects through specific molecular pathways rather than general anti‐inflammatory or antioxidant mechanisms.

This study underscores the significant antidepressant potential of geniposide, particularly through its ability to modulate astrocyte activity and reduce pyroptosis, thereby providing a novel therapeutic approach for depression. Beyond establishing geniposide as a potential antidepressant, this research reveals its mechanism of action involving specific molecular interactions, such as the regulation of lncRNAs and miRNAs. Furthermore, these findings may contribute to the development of new targeted treatments for depression, offering faster efficacy and fewer side effects, especially for patients who do not respond well to conventional antidepressant therapies.

Although geniposide has demonstrated significant potential as an antidepressant, this study has certain limitations. First, the findings are based on animal models, which cannot fully replicate the complexity and heterogeneity of human depression. Therefore, these results require further validation in human clinical trials. Second, the study primarily focused on the effects of geniposide on specific molecular pathways, potentially overlooking other relevant biological processes and systemic effects. Additionally, the long‐term safety and efficacy of geniposide were not assessed, which are critical factors for its translation into clinical practice.

Future research should address these limitations and expand upon these preliminary findings at multiple levels. Clinical trials are essential to evaluate the efficacy and safety of geniposide in patients with varying types and severities of depression. These trials should include dose‐response studies, safety assessments, efficacy evaluations, and long‐term follow‐ups. Furthermore, additional studies should explore other molecular and biological pathways affected by geniposide, examining how these pathways interact and contribute to the overall disease process. Moreover, future investigations should consider combining geniposide with existing treatment strategies, such as pharmacotherapy and cognitive behavioral therapy, to determine whether multimodal approaches can enhance therapeutic outcomes. By addressing these gaps through comprehensive research, geniposide and its related mechanisms could pave the way for novel treatments not only for depression but also for a broader range of neuropsychiatric disorders.

## 5 CONCLUSION

This study demonstrated that geniposide significantly alleviated depressive‐like behaviors in CUMS mice by upregulating the lncRNA Six3os1. Behavioral assessments, including the SPT, TST, OFT, FST, and MWM, showed that geniposide, particularly in the GP100 dose group, effectively restored normal behaviors in CUMS mice. Mechanistically, geniposide regulated the Six3os1/miR‐511‐3p/COL9A3 axis to inhibit the MAPK/NLRP3 signaling pathway, reducing astrocyte pyroptosis and improving hippocampal structure and function (Graphical Abstract).

This study is the first to reveal that geniposide exerts its antidepressant effects by upregulating lncRNA Six3os1, suppressing MAPK/NLRP3 pathway activation and inflammasome formation in a depression mouse model. These findings highlight lncRNA Six3os1 and miR‐511‐3p as promising therapeutic targets for depression. Clinically, geniposide, as a natural compound, demonstrates high safety and efficacy, highlighting its potential as a novel therapeutic agent for depression.

Although this study highlights the therapeutic potential of geniposide in depression, it has certain limitations. Because of experimental constraints, in vivo imaging techniques for astrocyte pyroptosis were not feasible; instead, primary astrocytes were isolated and cultured for ex vivo analysis. Moreover, the findings were validated solely in a CUMS mouse model and have not yet been confirmed in clinical samples. Given the complex etiology of depression, the efficacy of geniposide in different causes and stages of the disorder requires further investigation. Future research should encompass a broader range of animal models and clinical trials to validate these findings. Additionally, exploring other potential molecular targets and regulatory mechanisms associated with the Six3os1/miR‐511‐3p axis could lead to more effective treatments for depression.

## AUTHOR CONTRIBUTIONS

**Tianyu Zou**: Conceptualized and designed the study, performed experimental investigations, conducted data analysis, and drafted the original manuscript. **Cheng Mei**: Contributed to experimental design, validated results, curated critical datasets, and revised the manuscript for intellectual content. **Xiaoyu Liang**: Developed methodology, assisted in experiments, performed statistical analysis, and contributed to figure preparation. **Xiaolong Shang**: Provided technical resources, interpreted results, reviewed literature, and edited the manuscript. **Guoxiang Duan**: Supervised the project, acquired funding, oversaw data integrity, and approved the final version for submission. All authors critically reviewed the manuscript, approved the final draft, and agreed to be accountable for all aspects of the work.

## CONFLICT OF INTEREST STATEMENT

The authors declare no conflicts of interest.

## ETHICS STATEMENT

All animal experiments were approved by the Animal Ethics Committee of Shenzhen Luohu District Hospital of Traditional Chinese Medicine (No. 2023‐LHQZYYYXLL‐KY‐139).

## Supporting information

Supporting Information S1

## Data Availability

The datasets used or analyzed during this study are available from the corresponding author upon reasonable request.
