# Supplementary material for: Geniposide attenuates astrocyte pyroptosis in depression via long non‐coding RNA Six3os1‐mediated regulation of the miR‐511‐3p/COL9A3 axis and MAPK/NLRP3 signaling
Source: J Cell Commun Signal. 2025 Dec 2;19(4):e70043. doi: 10.1002/ccs3.70043 (PMC12670973; doi:10.1002/ccs3.70043)
Supplement: Supplementary file 1 — Supporting Information S1 [file CCS3-19-e70043-s001.docx]

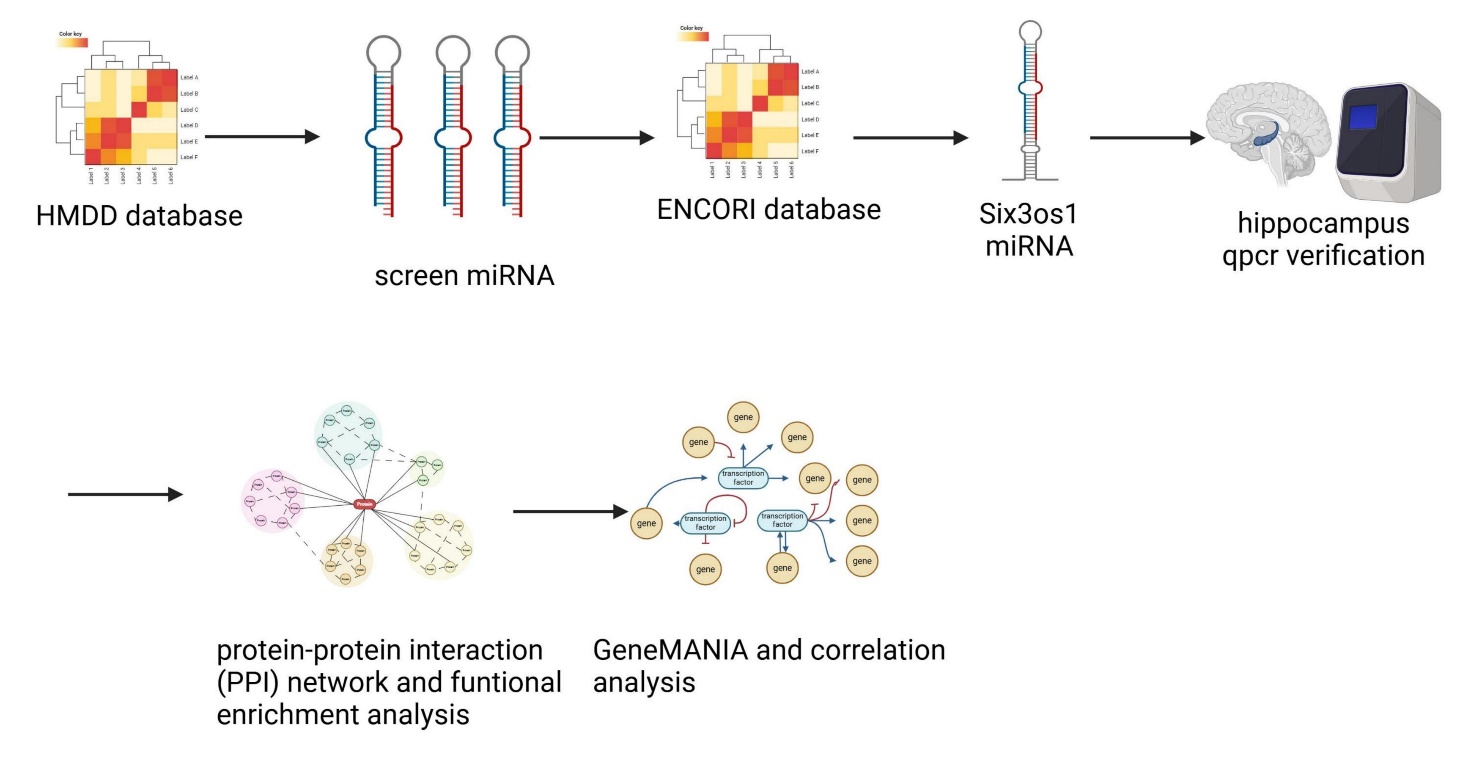


**FIGURE S1. Workflow for Screening and Validating Six3os1-Targeting miRNAs and Their Potential Functional Networks.**


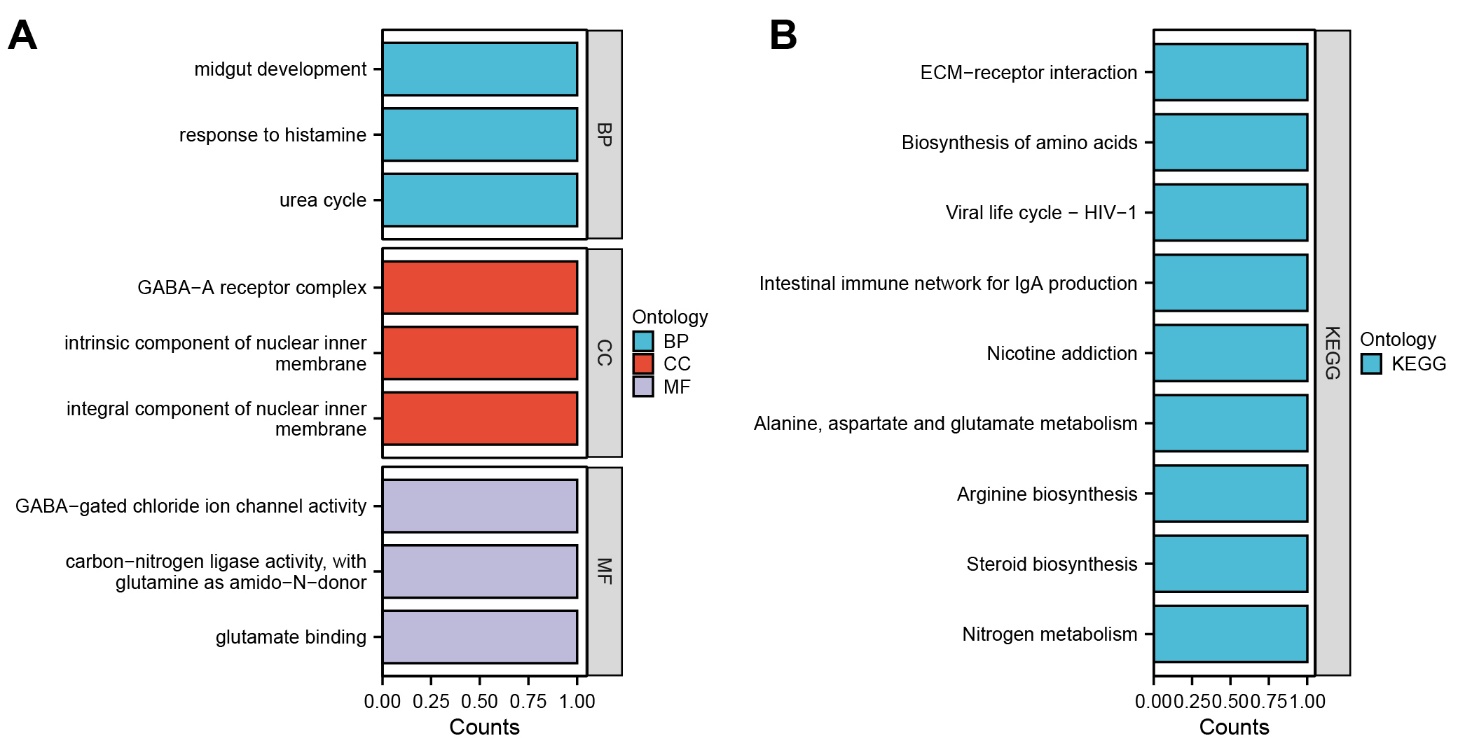


**FIGURE S2. GO and KEGG Functional Enrichment Analysis of Six Related Genes.**

Note: (A-B) GO and KEGG enrichment analysis results for the six related genes.


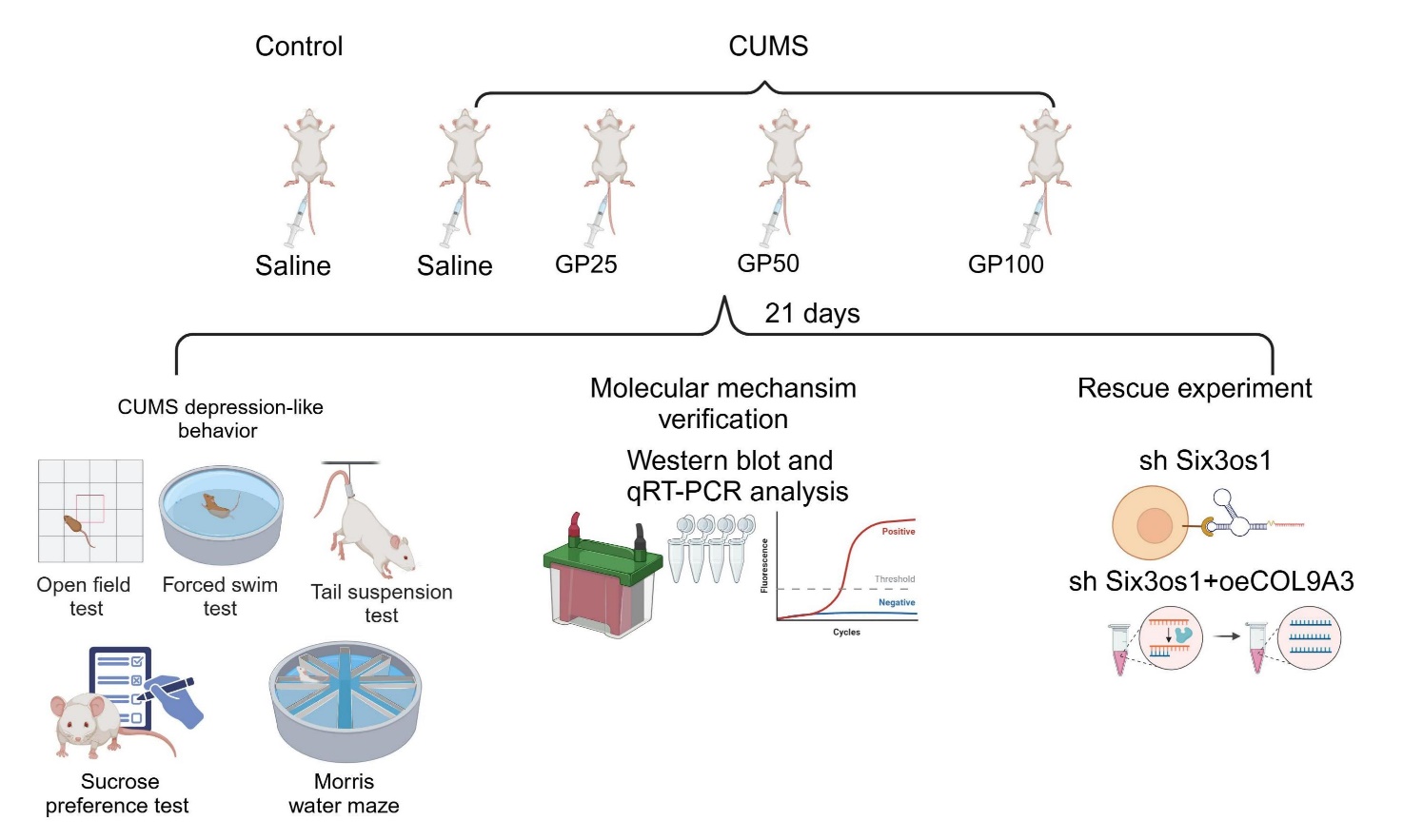


**FIGURE S3. Schematic Diagram of *In Vivo* Experimental Workflow for Geniposide Treatment in Mice.**


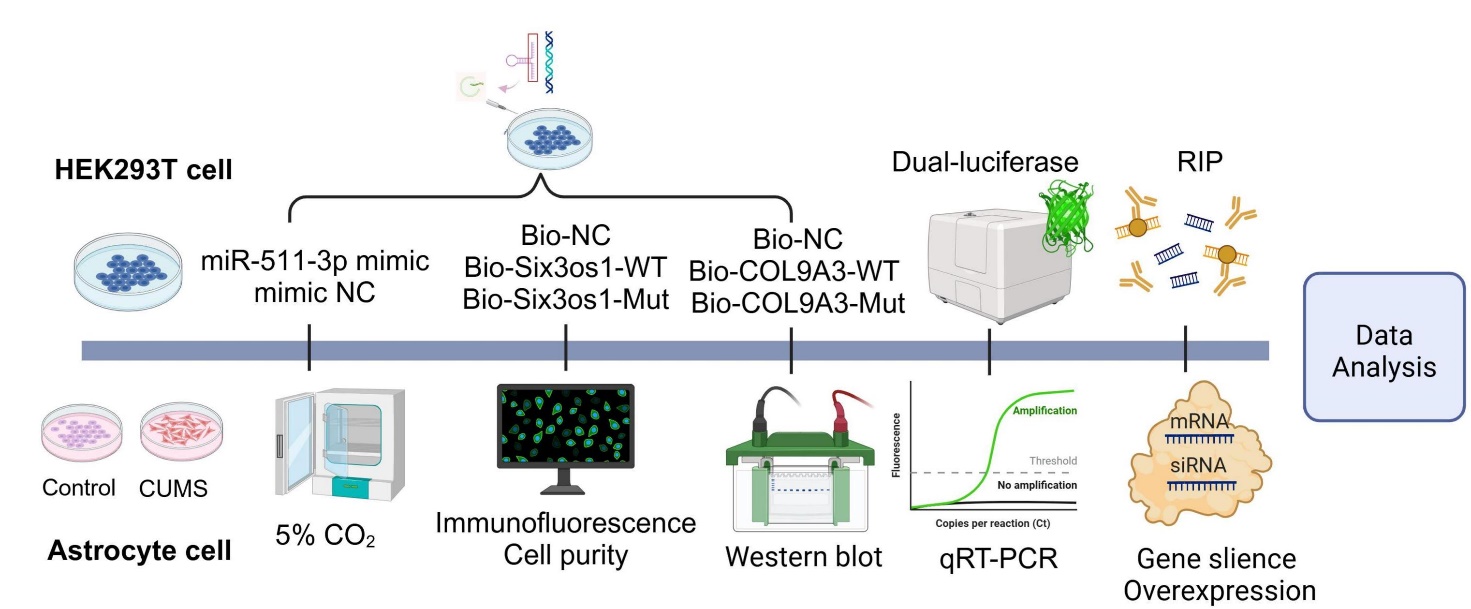


**FIGURE S4. Schematic Diagram of *In Vitro* Cellular Experimental Workflow.**


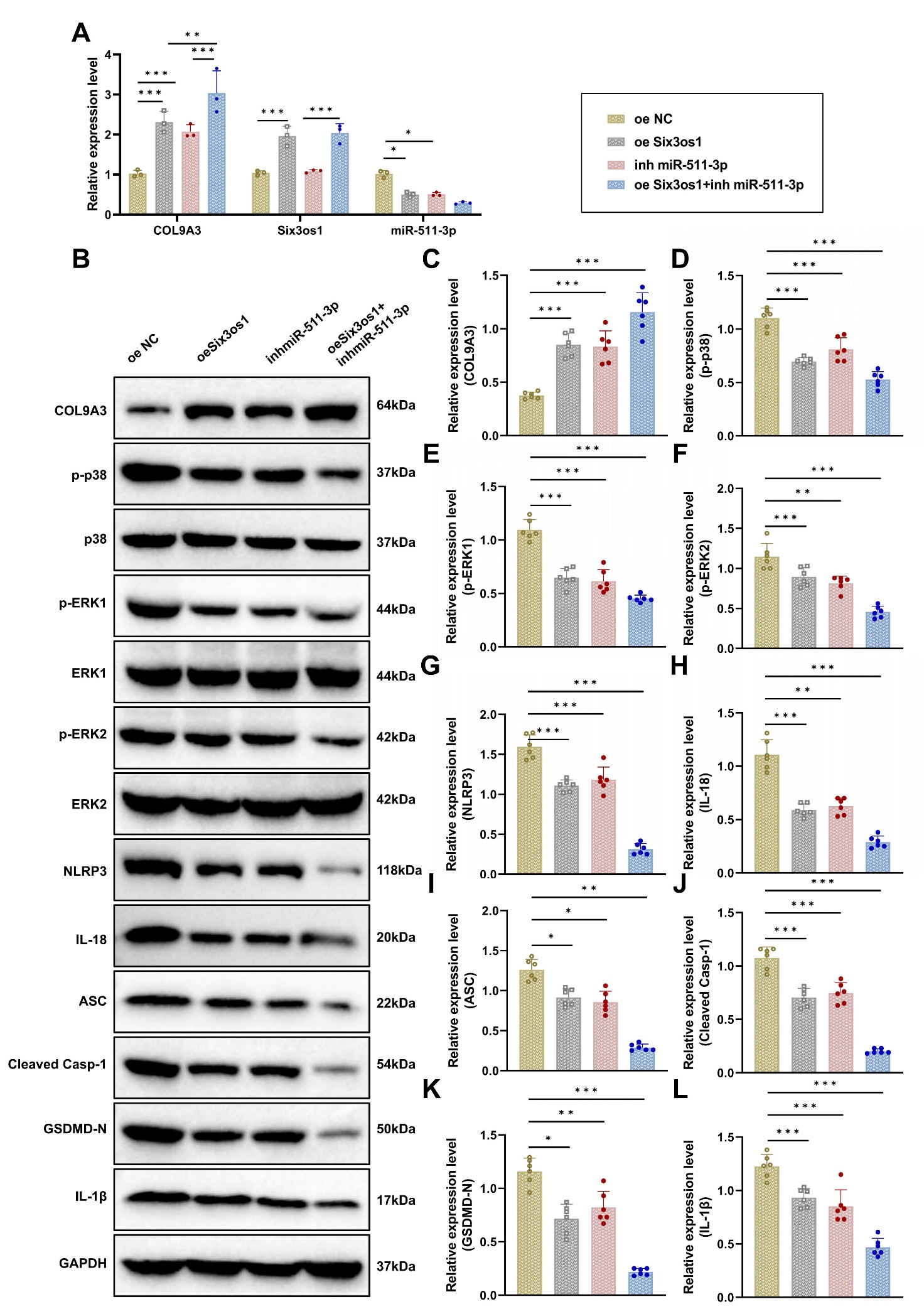


**FIGURE S5. Effects of Six3os1 Overexpression or miR-511-3p Inhibition on Key Protein Expression.**

Note: (A) qRT-PCR analysis of the effects of Six3os1 overexpression or miR-511-3p inhibition on the expression levels of COL9A3, Six3os1, and miR-511-3p in astrocytes from CUMS mice; (B) Western blot analysis of the effects of various treatments on COL9A3, p-p38, p-ERK1/2, NLRP3, IL-18, ASC, Cleaved Casp-1, GSDMD-N, and IL-1β protein expression in astrocytes from the CUMS model; (C-L) Relative protein expression levels for the following: (C) COL9A3; (D) p-p38; (E) p-ERK1; (F) p-ERK2; (G) NLRP3; (H) IL-18; (I) ASC; (J) Cleaved Casp-1; (K) GSDMD-N; (L) IL-1β. Data are presented as mean ± standard error. Statistical significance: **p <*0.05, ***p <*0.01, ****p <*0.001. N = 3.

**Table S1. RT-qPCR Primer Sequences.**

| **Genes (species)** | **Sequences (5’-3’)** |
| --- | --- |
| CXCR4 (mouse) | F: GACTGGCATAGTCGGCAATG |
|  | R: AGAAGGGGAGTGTGATGACAAA |
| LBR (mouse) | F: CTGCTTCCCATGAGGGTGAC |
|  | R: CCTTCTGCAACCTGCTTGTCT |
| CPS1 (mouse) | F: ACATGGTGACCAAGATTCCTCG |
|  | R: TTCCTCAAAGGTGCGACCAAT |
| VPS13B (mouse) | F: CAGTAAAGAGTCTCACGCTACAG |
|  | R: TGTTCCAGGGATGTCACCAGA |
| COL9A3 (mouse) | F: GGAATGCCGGGGTTCAAGG |
|  | R: AGTCCTCTTAATCCTCGTGGG |
| GABRB3 (mouse) | F: CTGCTGCCAATCTGGCTTTC |
|  | R: CGTAGCCTTTCAACAGCTTGTC |
| mmu-miR-124-3p | F: TAAGGCACGCGGTGAATGCC |
|  | R: Universal primer |
| mmu-miR-370-5p | F: CAGGTCACGTCTCTGCAGTT |
|  | R: Universal primer |
| mmu-miR-511-3p | F: AATGTGTAGCAAAAGACAGGAT |
|  | R: Universal primer |
| Mmu- miR-16-5P | F: TAGCAGCACGTAAATATTGGCG |
|  | R:Universal primer |
| Mmu-miR-370-3P | F:GCCTGCTGGGGTGGAACCTGGT |
|  | R:Universal primer |
| GAPDH (mouse) | F: AGGTCGGTGTGAACGGATTTG |
|  | R: TGTAGACCATGTAGTTGAGGTCA |
| U6 | F: GCTTCGGCAGCACATATACTAAAAT |
|  | R: CGCTTCACGAATTTGCGTGTCAT |

Note: F, forward; R, reverse.
